# An Ultra-High-Resolution Bending Temperature Decoupled Measurement Sensor Based on a Novel Core Refractive Index-Like Linear Distribution Doped Fiber

**DOI:** 10.3390/s22083007

**Published:** 2022-04-14

**Authors:** Yunshan Zhang, Yulin Zhang, Xiafen Hu, Dan Wu, Li Fan, Zhaokui Wang, Linxing Kong

**Affiliations:** 1 School of Aerospace Engineering, Tsinghua University, Beijing 100084, China; y.l.zhang@mail.tsinghua.edu.cn (Y.Z.); wangzk@tsinghua.edu.cn (Z.W.); 2 System Design Institute of Hubei Aerospace Technology Academy, Wuhan 430040, China; xiafenhu@163.com (X.H.); wudanbd@163.com (D.W.); 3 Beijing Tianji Space Technology Co., Ltd., Beijing 100084, China; fanli77@mail.tsinghua.edu.cn; 4 School of Opto-Electronic Information Science and Technology, Yantai University, Yantai 264005, China

**Keywords:** high resolution, bending sensor, temperature sensor, decoupling measurement

## Abstract

A high-resolution and high-sensitivity fiber optic sensor based on the quasi-linear distribution of the core refractive index is designed and fabricated, which enables decouple measurement of bending and of temperature. First, single-mode fiber doped with Al_2_O_3_, Y_2_O_3_, and P_2_O_5_ was drawn through a fiber drawing tower. The fiber grating was engraved on the fiber by a femtosecond laser. Modeling analysis was conducted from quantum theory. Experimental results show that the bending sensitivity of the grating can reach 21.85 dB/m^−1^, which is larger than the reported sensitivity of similar sensors. In the high temperature range from room temperature to 1000 °C, the temperature sensitivity was 14.1 pm/°C. The doped grating sensor can achieve high temperature measurement without annealing, and it has a distinguished linear response from low temperature to high temperature. The bending resolution can reach 0.0004 m^−1^, and the temperature resolution can reach 0.007 °C. Two-parameter decoupling measurement can be realized according to the distinctive characteristic trends of the spectrum. What’s more, the sensor exhibits excellent stability and a fast response time.

## 1. Introduction

With corresponding concepts such as the Internet of Things and smart cities, the sensing of information is an important content of the information age. As the perception layer of the information age, sensors are the entrance to the reception of massive amounts of information, and they are an essential foundation for the internet of everything. Sensing technology is evolving towards miniaturization, intelligence, integration, and passivity. The scope of physical perception is required to be more extensive, the means of information collection are more convenient, and the types of data acquisition are more diverse. 

The two physical parameters of temperature and curvature need to be evaluated in many applications, such as aerospace, petroleum energy, smart wearables, and industrial manufacturing [1]. After years of research, fiber bending and temperature sensors based on different principles have been reported. For example, the Mach–Zehnder interferometer [2], photonic crystal fiber [PCF] [3], four-core sapphire-derived fiber [4], seven core fiber [5], optical fiber laser [6], two-core fiber [7], three-core fiber [8], fiber Fabry–Perot cavity [9], Michelson interferometer [10], Sagnac Interferometer [11], long period fiber grating (LPFG) [12], fiber Bragg grating (FBG) [13], and capillary hollow-core fiber [14]. These sensors have their own unique advantages, but there are still many shortcomings. Interferometric sensors need to be fused multiple times, resulting in the reduced mechanical strength of the sensor [15,16]. Multi-core fiber and PCF are expensive to use [17]. Although the LPFG sensor has high sensitivity, it usually analyzes the data through a transmission spectrum, and it is therefore difficult to reuse the optical fiber sensor network [18]. When an FBG sensor is used to evaluate bending and temperature, it has low sensitivity and it cannot distinguish measurement [19]. In terms of temperature measurement, the sapphire grating sensor can measure very high temperatures. However, the cost is high and the manufacturing technology is not mature enough [20]. The regenerative grating can measure high temperature, but it requires a long period of high temperature annealing treatment, after which the grating area will be very brittle [21].

Therefore, in order to address the above problems, we propose a FBG sensor engraved by femtosecond laser on a new doped fiber. First, we designed and drew a new type of fiber doped with Al_2_O_3_, Y_2_O_3_, and P_2_O_5_. The core refractive index of the new optical fiber is quasi-linear distribution. The FBG is engraved on the fiber by the femtosecond laser. The grating model is analyzed by quantum optics theory and refractive index perturbation theory. Bending and temperature experiments were performed on this new type of grating. Experimental results found that the grating exhibited very high bending sensitivity. The grating can achieve continuous temperature measurement without long-term high temperature annealing. The grating spectral energy is sensitive to bending, and the wavelength is sensitive to temperature. It exhibits unique spectral properties for two different physical quantities. The sensor measurement system has ultra-high curvature and temperature resolution. 

## 2. Fabrication and Theoretical Analysis

The purpose of traditional optical fiber doping materials is to obtain the refractive index difference between the core and the cladding. However, the purpose of doped oxide materials in this manuscript is to enhance the temperature resistance properties of the fiber, while modulating the refractive index distribution of the core. The melting point of Al_2_O_3_ is 2054 °C. Additionally, Al_2_O_3_ has the characteristics of a small specific surface area, uniform particle size, easy dispersion, high hardness and good insulation performance, which makes the fabrication of optical fiber preform and the drawing of optical fiber easier to control. Meanwhile, Al_2_O_3_ has the characteristics of high mechanical strength, strong wear resistance, and heat shock resistance, which makes the sensor more robust. It holds the characteristics of colorless and transparent, high light transmittance and high refractive index [22]. The melting point of Y_2_O_3_ is 2439 °C. Below 2200 °C, Y_2_O_3_ is a cubic phase without birefringence [23,24]. It holds the characteristics of corrosion resistance, wide optical transparency, and good physicochemical properties.

**Figure 1:**
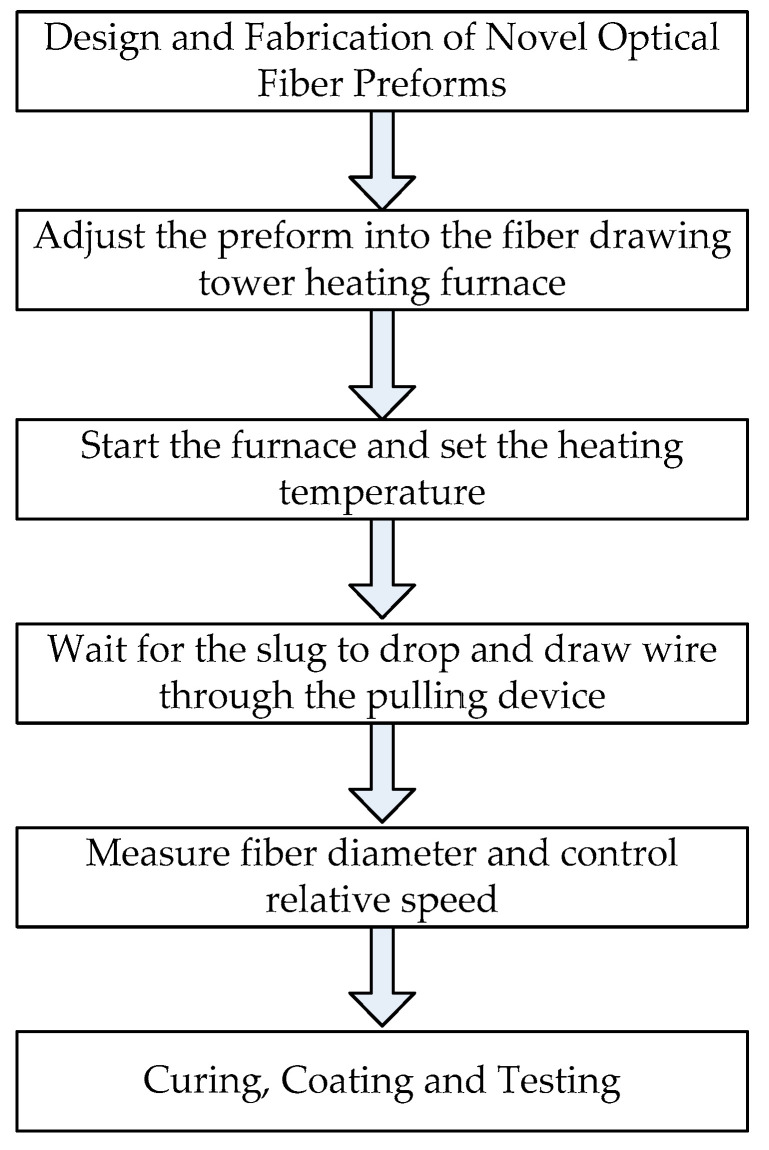
Schematic diagram of new optical fiber manufacturing.

A new type of optical fiber can be obtained by drawing the optical fiber preform through the optical fiber drawing tower. The high-temperature resistance furnace of the drawing tower is heated to 2300 °C, so that the cone of the optical fiber preform is melted to form a droplet like material head. After controlling the thinning of the optical fiber preform, it goes through an annealing device and a cooling system in turn. The dimension parameters of optical fibers are supervised online in real time. Finally, the bare fiber is coated and cured by means of a coating device. Figure 1 presents a schematic diagram to reinforce the description of the novel fiber fabrication.

Table 1 presents the measured dimensional parameters and numerical apertures of the novel fiber.

The doped optical fiber core was analyzed by electron probe micro analysis (EPMA). The EPMA enables qualitative and quantitative analysis of chemical composition of microscopic regions in fiber. The mass percentage of different doping can be achieved by fully automatic area scanning analysis. Its core contains 4.9 wt.%Y_2_O_3_, 3.2 wt.%Al_2_O_3_, and 2.3 wt.% P_2_O_5_. It is the most sensitive optical fiber measurement technology available in the testing wavelength range of 375 nm~2000 nm, which can measure the refractive index, stress, and geometry of any type of fiber. Table 2 gives the detailed parameters of FRIMA-IFA-100-IR.

**Figure 2:**
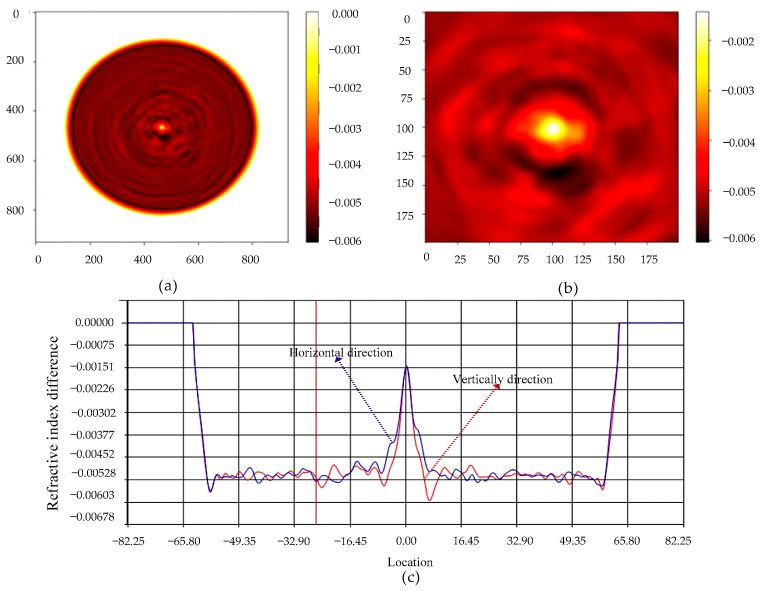
New fiber parameters, (**a**) Optical fiber cross-section refractive index profile; (**b**) Fiber Core Refractive Index Profile; (**c**) Refractive index difference in horizontal direction and in vertical direction of optical fiber.

Refractive index measurements of fiber cross-sections are set out in Figure 2. The maximum refractive index is 1.464, and the cladding refractive index is significantly less than the core index. The abscissa and the ordinate represent the number of measurement sampling points in Figure 2a. Locally amplified refractive index profile measurements were performed on the fiber core region and the results are presented in Figure 2b. The point of maximum refractive index of the fiber is located in the center of the fiber core. Its corresponding core refractive index presents a non-uniform distribution of the point spread function. The refractive index difference in the horizontal direction and in the vertical direction of the fiber were measured, and the results are depicted in Figure 2c. From the results, it is found that the refractive index distribution of the fiber cladding is relatively uniform, but the core refractive index almost implies a linear decrease. In addition to this, the core has a small amount of jitter in the horizontal and in the vertical refractive index profiles. When light is transmitted in the new optical fiber structure, the mode of the light field will change, causing the total reflection angle of light to become smaller when light is transmitted in the optical fiber. When the optical fiber is bent, it will cause part of the optical energy to be coupled into the optical fiber cladding. Taking advantage of this property of the new type of optical fiber, the sensor can be used for bending measurements by demodulating the energy. 

Fiber gratings were engraved by direct writing using the femtosecond laser micromachining system. Femtosecond lasers are characterized by ultra-short pulse widths and ultra-high peak powers. When the femtosecond laser interacts with the fiber material, nonlinear ionization phenomena such as nonlinear photoionization and avalanche ionization are mainly generated. The high-energy femtosecond laser acts on the fiber to cause the formation of internal defects or local shrinkage of the material. It densifies the material and causes localized melting of the fiber core, resulting in refractive index modulation for permanent material damage. What’s more, the avalanche ionization effect is generated and the fiber material continuously absorbs the laser irradiation energy. Eventually, the electron plasma in the laser focus area will increase to a certain concentration value, and more intense light absorption will occur, resulting in permanent refractive index modulation in the irradiated area.

**Figure 3:**
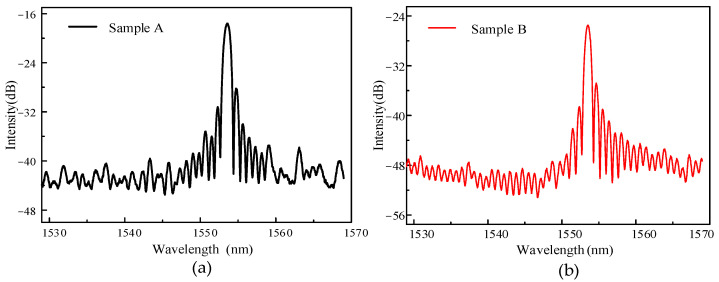
Reflection spectra of samples with different gratings. (**a**) Sample A; (**b**) Sample B.

The center wavelength of the femtosecond laser was 800 nm. The repetition rate was 200 kHz. The pulse width was 200 fs, and the energy range was tunable from 0 to 3 W. A laser energy attenuation system was used to adjust the laser output energy, and finally the refractive index was modulated on the new optical fiber by focusing on a high-magnification microscope objective. The processing position and the modulation effect of the front end of the laser imaged and observed by CCD. In the process of preparing the grating, the optical fiber was fixed on the three-dimensional displacement stage through the adsorption clamp platform. The computer terminal controlled the three-dimensional stage to make the fiber move at a specific speed and in a specific direction. The reflection spectrum appeared in real time through the demodulator. Figure 3 is the grating spectrum engraved under the above femtosecond laser parameters. It can be seen from Figure 2 that the grating reflection spectrum had a higher contrast and a lower noise. The center wavelength of the sample A grating was 1533.58 nm. The grating period was 1.2 μm. The number of lines engraved by the laser was 5000, and the corresponding grating length was 6 mm. The center wavelength of the sample B grating was 1533.58 nm. The grating period was 1.2 μm. The number of lines engraved by the laser was 3000, and the corresponding grating length was 3.6 mm. Figure 3a,b show the reflection spectra of two different grating samples with lengths of 6 mm and 3.6 mm, respectively. The length of the sample A grating was greater than that of the sample B grating. The period of the sample grating was the same. The difference was that the length of engraving was different under the same period. 

This manuscript presents an analogous approach to theoretical analysis in which the micromachining of optical fibers by femtosecond lasers is considered as a perturbation of the refractive index of the fiber core. It is proposed to deduce the theory of grating coupled mode by establishing the perturbation theory applicable to Maxwell’s equations. The main idea of modeling is as following: introduce the state vector

(1) $$|\psi \rangle =\left(\begin{array}{c} \vec{E}\\ j\vec{H} \end{array}\right)$$

and assume that the electromagnetic field changes with time in a relationship of $e^{j\omega t}$. So, Maxwell’s equation can be expressed as:

(2) $${\hat{L}}_{t}|\psi \rangle +{\hat{L}}_{z}|\psi \rangle -\frac{\omega }{c}\hat{W}|\psi \rangle =0$$

where

(3) $${\hat{L}}_{t}=\left(\begin{array}{cc} 0 & \nabla_{t}\times \\ \nabla_{t}\times & 0 \end{array}\right),{\hat{L}}_{z}=\left(\begin{array}{cc} 0 & \frac{\partial }{\partial z}{\vec{e}}_{z}\times \\ \frac{\partial }{\partial z}{\vec{e}}_{z}\times & 0 \end{array}\right),\hat{W}=\left(\begin{array}{cc} \overset{↔}{\varepsilon } & 0\\ 0 & \overset{↔}{\mu } \end{array}\right)$$

For an unperturbed fiber, the state vector corresponding to the propagation constant analysis $\beta_{k}$ is ${|\psi \rangle }_{k}=e^{j\beta_{k}z}|\psi_{k}(x,y)\rangle$. $|\psi_{k}(x,y)\rangle$ is the transverse eigenfunction of the state vector, which satisfies the eigen equation

(4) $$\left(-{\hat{L}}_{t}+\frac{\omega }{c}{\hat{W}}_{0}\right)|\psi_{k}\rangle =\beta_{k}{\hat{\Gamma }}_{z}|\psi_{k}\rangle$$

(5) $${\hat{\Gamma }}_{z}=j\left(\begin{array}{cc} 0 & {\vec{e}}_{z}\times \\ {\vec{e}}_{z}\times & 0 \end{array}\right),{\hat{W}}_{0}=\left(\begin{array}{cc} \varepsilon_{u} & 0\\ 0 & 1 \end{array}\right)$$

when the optical fiber core is perturbed by the femtosecond laser, the perturbation form is set as

(6) $${\hat{W}}_{\delta }=\hat{W}-{\hat{W}}_{0}$$

the electromagnetic field propagating in the fiber is the superposition state of the eigenmodes in the undisturbed fiber, namely

(7) $$|\psi \rangle =\sum_{k}a_{k}(z)e^{j\beta_{k}z}|\psi_{k}\rangle$$

According to the time-dependent perturbation theory of quantum mechanics, the superposition state |ψ〉 can evolve according to the following equation

(8) $${\hat{L}}_{z}|\psi \rangle =\left(-{\hat{L}}_{t}+\frac{\omega }{c}{\hat{W}}_{0}\right)|\psi \rangle +\frac{\omega }{c}{\hat{W}}_{\delta }|\psi \rangle$$

Using the orthonormalization condition, the coupled mode equation for the mode amplitude can be obtained as follows

(9) $$\frac{\partial }{\partial z}a_{j}(z)=j\sum_{k}a_{k}(z)e^{j(\beta_{k}-\beta_{j})z}\langle \psi_{j}|{\hat{W}}_{\delta }|\psi_{k}\rangle$$

where ${\hat{W}}_{\delta }$ is related to the perturbation of the dielectric constant. If the perturbation form of the dielectric constant caused by the refractive index modulation is found, the corresponding perturbation coupled mode equation can be obtained.

## 3. Experiment and Discussion

**Figure 4:**
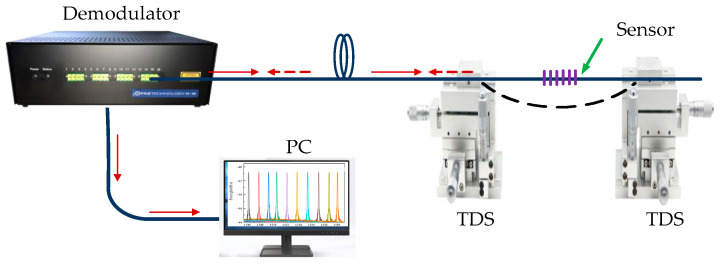
Bending experiment test device system.

Figure 4 is a curvature experiment measurement device system. One end of the grating was connected to a high-precision demodulator (FAZT), and its wavelength resolution was 0.1 pm. The demodulator is connected to the computer, and the reflection spectrum information of the grating can be presented in real time on the computer’s software. The grating bending region is encapsulated in elastic capillaries to prevent the grating from breaking due to stress concentration. The elastic capillary is fixed on the two three-dimensional translation stages (TDSs) by the fiber holder, and the curvature of the sensor can be modified by changing the distance between the two translation stages. The grating area is always centered on both stages while preventing twisting of the sensor. When the fiber grating region is bent, its curvature function can be expressed as

(10) $$\sin\frac{L}{2R}=\frac{D}{R}$$

where R is the radius of curvature of the fiber grating; L represents the initial distance between the two TDSs; and D is the absolute value of the travel distance between the TDSs.

**Figure 5:**
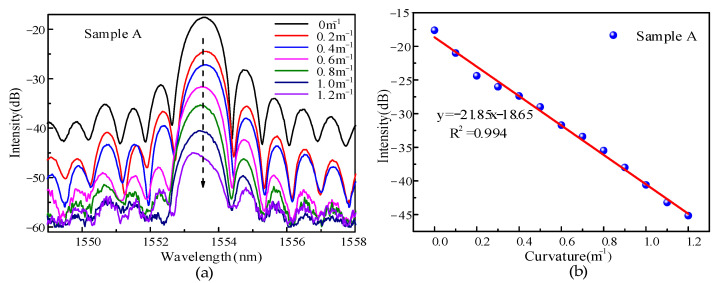
(**a**) Reflection spectrum of sensor under different curvature; (**b**) Bending fitting curve.

Bending experiments were carried out on the novel grating sample A, and the results are shown in Figure 5a. The center wavelength of the sample A grating was 1533.58 nm. The grating period was 1.2 μm. The number of lines engraved by the laser was 5000, and the corresponding grating length was 6 mm. Ambient temperature remained constant when measuring curvature. The reflection spectra under different curvature values were recorded. The experimental results clearly reveal that the reflection spectral response of the grating was very sensitive with the change of curvature. Further analysis according to the changing characteristics of the spectrum shows that the energy of the resonant spectrum changes very rapidly to bending, but the wavelength is hardly affected. Through the experimental consequences, the potential trend of central spectral energy with curvature is analyzed again. The energy of the reflected spectrum drops sharply with increasing curvature. This is attributed to the fact that most of the light transmitted in the grating region are coupled into the fiber cladding with increasing curvature. The energy of the sensor changed by 27.5 dB when the curvature range increased from 0 to 1.2 m^−1^. A linear fit was performed on a large number of data points, and the fitting results are shown in Figure 5b. The bending sensitivity of the grating was −21.85 dB/m^−1^ and the linearity amounted to 0.994, which is very convenient for signal demodulation. Since the energy resolution of the sensor signal demodulator was 0.01 dB, the bending resolution of the sensor can be obtained by calculation to be 0.0004 m^−1^. A large number of experimental measurements were carried out, all exhibiting similar sensing properties. Therefore, in the following figures we give the specific experimental results of sample A to represent the sensing characteristics of this type of sensor.

**Figure 6:**
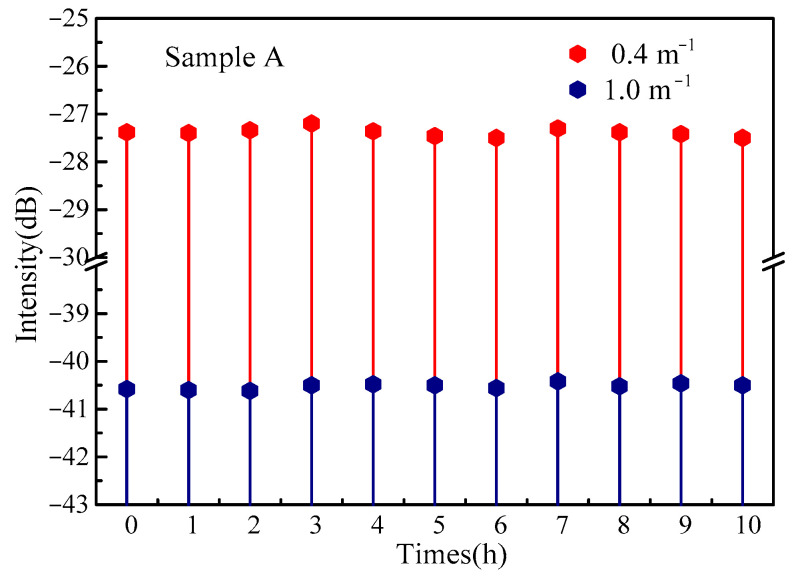
Bending stability test of sensor.

The stability of the sensor was checked for ten hours when the curvature was 0.4 m^−1^ and 1.0 m^−1^, respectively. The experimental results are shown in Figure 6. The sensor demonstrates exceptional long-term stability. In the long-time measurement process, the maximum fluctuation of reflected spectral energy was 0.3 dB. Considering bending sensitivity of the grating, the curvature fluctuation of the bending was 0.014 m^−1^.

**Figure 7:**
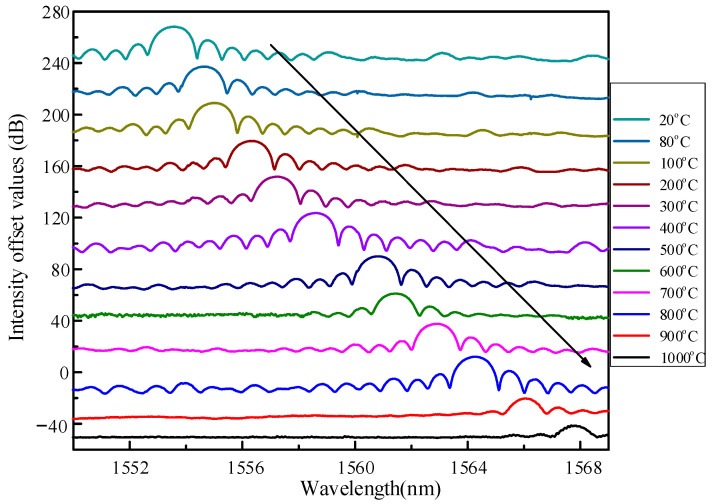
Grating reflection spectrum at different temperatures.

The temperature characteristics of fiber gratings doped with high temperature resistant materials were checked. The heating furnace adopted high-quality high alumina polycrystalline ceramic fiber, which holds the characteristics of rapid heating resistance, stability, and uniform heating in the heating area. The maximum working temperature of the tubular furnace was 1100 °C, and the temperature control accuracy was 1 °C. The length of the heating zone was 300 mm. The temperature measurement area was calibrated by a soaring temperature thermocouple contact probe. Then, place the grating area in a free straightening state in a heating furnace and raise the temperature from room temperature to 1000 °C, and the room temperature is 20 °C; record the spectral data every 100 °C, and stabilize the temperature at the recording point for half an hour to heat it evenly. Figure 7 records the spectrum of grating at different temperatures. In Figure 6, we still give the experimental measurement results of sample A. The sensing properties of this novel sensor are represented by sample A. 

With the increase of temperature, the wavelength of the grating drifts in the long wave direction. The effect of temperature on the wavelength of fiber grating includes three aspects. The thermal expansion effect of the fiber causes the grating grid spacing to change. The optical fiber thermo-optic effect changes the refractive index of the fiber grating. The elastic-optic effect caused by the thermal stress inside the fiber makes the core diameter of the fiber change. The total effect of temperature on FBG wavelength drift is:

(11) $$\frac{\Delta \lambda_{B}}{\lambda_{B}}=(\alpha +\zeta )\Delta T$$

where $\lambda_{B}$ is the center wavelength of the grating; α is the thermal expansion coefficient of the fiber material, and its value is greater than zero; and ζ is the thermo-optic coefficient of the fiber material, and its value is greater than zero. So, $\Delta \lambda_{B}$ will be a value greater than zero when ΔT is greater than zero. From this, it can be known that the grating wavelength will drift to the long wave direction as the temperature increases.

**Figure 8:**
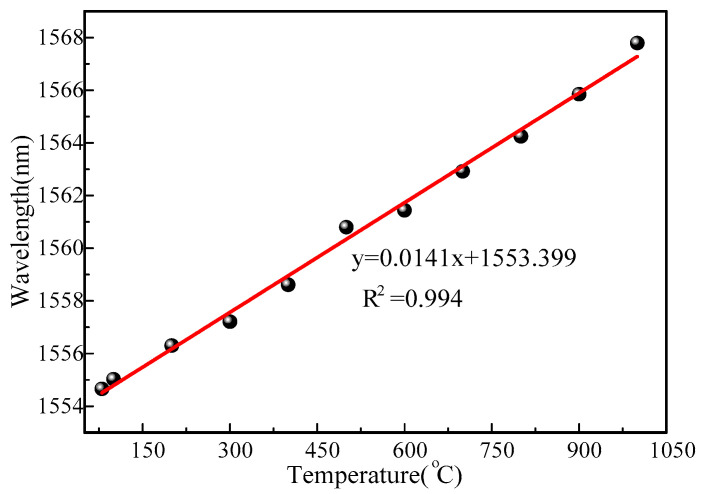
Temperature fitting curve.

The energy of the reflection spectrum hardly changed in the temperature range before 850 °C. When the ambient temperature exceeds 850 °C, the internal stress of the fiber will be unevenly distributed, causing changes in the effective period and in the effective refractive index of the grating. Finally, the original refractive index modulation of the fiber grating is changed. Refractive index modulation recombines and weakens resulting in a reduction in reflected spectral energy. The grating reflectance spectra showed very high extinction ratios even without prolonged annealing during the heating process. At present, the reported grating sensors that can measure high temperature need a long regeneration process [21]. The resonant wavelength is linearly fitted, as shown in Figure 8. The sensitivity of the sensor was as high as 14.1 pm/°C in the range from room temperature to 1000 °C, and the linearity could reach 0.994. The wavelength resolution of the demodulator was 0.1 pm. The temperature resolution of the sensor could be obtained by calculation to be 0.007 °C.

**Figure 9:**
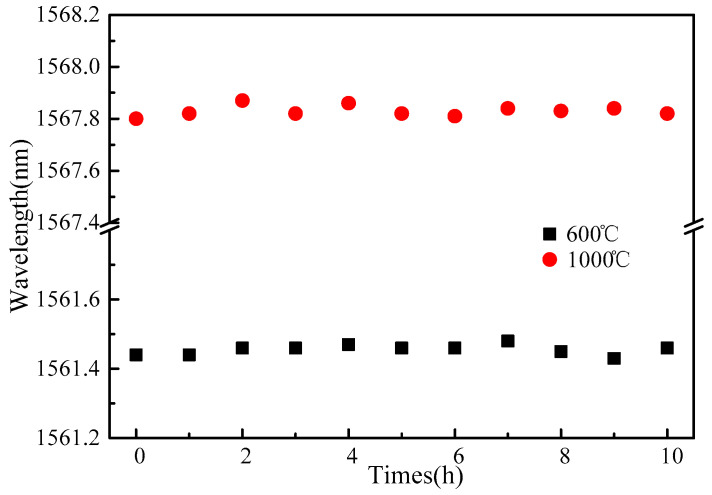
Temperature stability experiment.

The temperature stability of the FBG was continuously monitored for a long time. The output spectrum of the sensor was monitored for 10 h when the temperature was 600 °C and 1000 °C, respectively. The implementation results are shown in Figure 9. The maximum wavelength fluctuation of the reflection spectrum was 18 pm, and the corresponding temperature fluctuation did not exceed 1.2 °C. This fluctuation had little effect compared with the absolute value of the measured temperature. 

**Figure 10:**
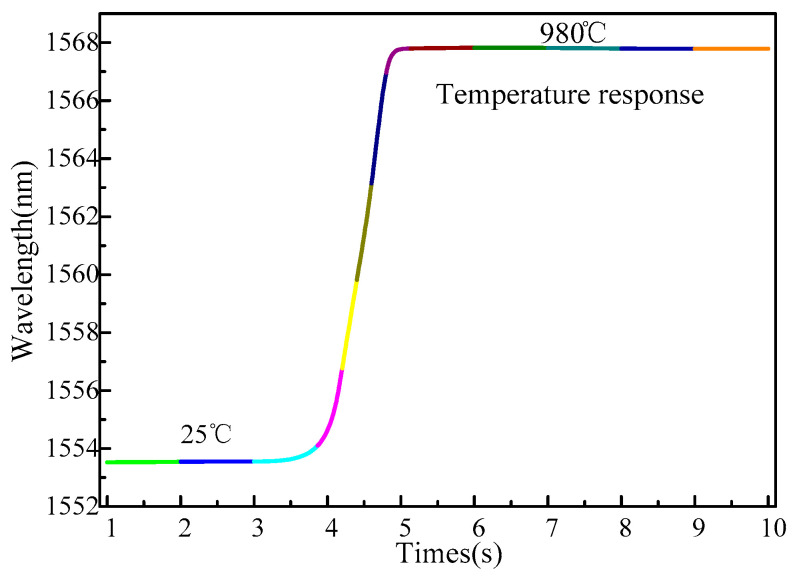
Temperature response time.

There have been reports about the high sensitivity of fiber grating temperature sensors, but there are few reports about its response time characteristics. The response time of the sensor to temperature is an important parameter of the sensor’s sensing index. Therefore, a response time test experiment was conducted: instantly switch the sensor from 25 °C to 980 °C high temperature environments; the wavelength signal of the sensor is perceived by the demodulation system, and its sampling frequency is 1 kHz; as shown in Figure 10, the response time of the sensor is 0.6 s for the temperature jump of 955 °C. Since manual switching will cause a specified delay when the sensor switches between two different temperature environments, the actual response speed of the temperature sensor should be faster. The influence of sensor measurement error caused by inconsistent sensor response is eliminated. The response of the traditional platinum resistance temperature sensor is more than 10 s. Although the response time of the thermistor can be very short, the current is difficult to control during the test, and it is often very large, which will cause substantial measurement errors. The extra fiber sensor shows an extremely fast measurement response. 

Table 3 summarizes the comparison of sensing characteristics of different types of sensors. It can be found from the table that the sensor designed in this paper exhibits excellent sensing characteristics.

The resolution comparison between the sensor in the paper and the same type of fiber optic sensor is given in Table 4. It is obvious from the table that the sensor designed in this paper exhibits a very high measurement resolution.

## 4. Conclusions

In this paper, a new type of optical fiber based on different doping materials is designed first, and the refractive index of the core of the optical fiber exhibits a quasi-linear distribution. The ratio and the effective refractive index of different doping materials for the new optical fiber are analyzed. Fiber gratings were engraved on the new fiber by femtosecond laser. The grating model was analyzed by quantum optics theory and refractive index perturbation theory. Bending and temperature experiments were performed on the sensor. The bending experiment results show that the sensitivity of the sensor is as high as 21.85 dB/m^−1^ in the curvature range of 0 to 1.2 m^−1^, and its linearity is 0.994. Stability experiments show that the long-term experimental bending fluctuation measurement error is 0.014 m^−1^. The temperature experiment results show that the grating sensitivity is 14.1 pm/°C in the temperature range from 20 °C to 1000 °C, and its linearity is 0.994. At the same time, the sensor shows excellent stability. The sensing system has extremely high bending and temperature measurement resolution. The grating does not require long-term annealing to enable high temperature measurements. The temperature response time of the sensor is 0.6 s for the temperature jump of 955 °C. Due to excellent sensing properties, it has important potential applications in extreme conditions.

## Figures and Tables

**Table 1:** Parameters of novel high temperature resistant doped fiber.

Core Composition	Cladding Diameter	Core Diameter	Coating Diameter	Numerical Aperture
SiO_2_-Al_2_O_3_-Y_2_O_3_-P_2_O_5_	125 μm	10 μm	252 μm	0.2

**Table 2:** Measured parameters of FRIMA-IFA-100-IR.

Refractive Index Measurement Accuracy	Spatial Resolution	Measuring Concentricity Error	Measure Core Out-of-Roundness Error
±0.0001	500 nm	±200 nm	±0.4%

**Table 3:** Comparison of sensing characteristics of different types of sensors.

Sensors Structure	Bending Sensitivity	Range	Temperature Sensitivity	Range	Distinguishing Measurement	Reference
Four-Core Sapphire-Derived Fiber	4.5 dB/m^−1^	0.4-2.5 m^−1^	/	/	No	[4]
Seven-core FBG	7.2 dB/m^−1^	0–1.0 m^−1^	12 pm/°C	35–215 °C	No	[5]
Optical fiber laser	1.04 nm/m^−1^	0.8–2.0 m^−1^	/	/	No	[6]
Two-core MZI	−6.18 nm/m^−1^	0–0.98 m^−1^	31 pm/°C	30–70 °C	No	[7]
Three core LPFG	3.23 nm/m^−1^	0–0.58 m^−1^	54 pm/°C	30–80 °C	No	[8]
Concave-lens-like LPFG	32.78 nm/m^−1^	0–2.08 m^−1^	54 pm/°C	30–90 °C	No	[12]
SMF-FCF-SMF	−18.75 nm/m^−1^	0.042–0.163 m^−1^	74 pm/°C	30–80 °C	Yes	[15]
Off-axis written FBG	−1.25 dB/m^−1^	0–1.1 m^−1^	10.8 pm/°C	23.5–60 °C	No	[19]
This work	21.85 dB/m^−1^	0–1.2 m^−1^	14.1 pm/°C	20–1000 °C	Yes	

**Table 4:** Resolution comparison between different types of sensors.

Sensor Structure	Bending Resolution	Temperature Resolution	Reference
Four-Core Sapphire-Derived Fiber	0.008 m^−1^	\	[4]
Seven-core FBG	0.001 m^−1^	0.08 °C	[5]
Optical fiber laser	0.004 m^−^^1^	\	[6]
Two-core fiber taper	0.003 m^−^^1^	0.3 °C	[7]
Three-core LPFG	0.006 m^−^^1^	0.35 °C	[8]
Concave-lens-like LPFG	0.0006 m^−^^1^	0.32 °C	[12]
graded index multimode fiber	\	0.11 °C	[18]
Off-axis	0.039 m^−^^1^	\	[19]
This work	0.0004 m^−1^	0.007 °C	

## Data Availability

Not applicable.
